# Recent Advances in the Use of Molecular Analyses to Inform the Diagnosis and Prognosis of Patients with Polycythaemia Vera

**DOI:** 10.3390/ijms22095042

**Published:** 2021-05-10

**Authors:** Ruth Stuckey, María Teresa Gómez-Casares

**Affiliations:** Hematology Department, Hospital Universitario de Gran Canaria Dr. Negrín, 35019 Las Palmas, Spain; rstuckey@fciisc.es

**Keywords:** myeloproliferative neoplasms, molecular analysis, risk stratification, targeted therapy, personalized medicine

## Abstract

Genetic studies in the past decade have improved our understanding of the molecular basis of the *BCR-ABL1*-negative myeloproliferative neoplasm (MPN) polycythaemia vera (PV). Such breakthroughs include the discovery of the *JAK2*V617F driver mutation in approximately 95% of patients with PV, as well as some very rare cases of familial hereditary MPN caused by inherited germline mutations. Patients with PV often progress to fibrosis or acute myeloid leukaemia, both associated with very poor clinical outcome. Moreover, thrombosis and major bleeding are the principal causes of morbidity and mortality. As a result of increasingly available and economical next-generation sequencing technologies, mutational studies have revealed the prognostic relevance of a few somatic mutations in terms of thrombotic risk and risk of transformation, helping to improve the risk stratification of patients with PV. Finally, knowledge of the molecular basis of PV has helped identify targets for directed therapy. The constitutive activation of the tyrosine kinase JAK2 is targeted by ruxolitinib, a JAK1/JAK2 tyrosine kinase inhibitor for PV patients who are resistant or intolerant to cytoreductive treatment with hydroxyurea. Other molecular mechanisms have also been revealed, and numerous agents are in various stages of development. Here, we will provide an update of the recent published literature on how molecular testing can improve the diagnosis and prognosis of patients with PV and present recent advances that may have prognostic value in the near future.

## 1. Introduction

According to the World Health Organization’s (WHO) classification, polycythaemia vera (PV) is classified as a myeloproliferative neoplasm (MPN), a group of chronic disorders characterized by the clonal proliferation of one or more blood cell lines in the myeloid lineage that also includes essential thrombocythemia (ET), primary myelofibrosis (PMF), chronic myeloid leukaemia, chronic neutrophilic leukaemia, chronic eosinophilic leukaemia-not otherwise specified, and MPN unclassifiable [1,2]. Of these, PV is particularly prevalent, affecting approximately 22/100,000 people [3]. Nevertheless, PV is classified as a “rare disease” by Orphanet, the European-wide database supported by the European Commission (www.orpha.net, accessed on 10 October 2020).

PV is a *BCR-ABL1*-negative MPN characterized by the clonal proliferation of hematopoietic progenitor cells, resulting in an increased production of morphologically normal red blood cells, white blood cells and platelets, although erythrocytosis most often predominates. The disorder is most common in those aged 50–70 years, with an incidence that increases with older age. Patients diagnosed with PV have a shorter survival compared to the age-matched general population, with a median survival of 13.5 years [2], due to an increased predisposition for thrombosis as well as transformation to myelofibrosis (post-PV myelofibrosis, estimated to occur in 10% of patients with PV) or acute myeloid leukaemia (AML, estimated to occur in 15% of patients with PV) [3]. The key goals of treatment are to prevent thrombosis and bleeding while relieving PV-related symptoms (such as pruritus, headaches, blurred vision, dizziness, erythema, and fatigue), with the latter reported by 33% of the 380 patients with PV who answered the MPN Landmark survey to be the symptom they most wanted to resolve [4].

Rapidly evolving molecular techniques, in particular next-generation sequencing technologies (NGS), have improved our understanding of the molecular basis of MPNs including PV (reviewed in [5]). Genetic studies are increasingly revealing the presence of somatic mutations and/or gene expression changes that can help refine the diagnosis or prognosis of PV. As a result, the integration of molecular information into clinical decision-making algorithms may improve the risk stratification of patients with this disorder.

This review will explore how recent advances in molecular testing can aid the management of PV in the clinic for a more accurate diagnosis and prognosis, such as for a more informed prediction of the risk of developing thrombosis and progression to secondary MF or AML.

## 2. Diagnosis

Diagnosis of PV is made according to the 2016 WHO criteria and is based on the assessment of several clinical, haematological and molecular features, including serum haemoglobin level, *BCR-ABL1*-negativity, and *JAK2* mutation status [1,2]. Here, we will focus on how the molecular testing of genetic alterations can inform a more precise diagnosis of PV and recommend readers to two comprehensive reviews for general guidelines on the diagnosis and management of patients with PV [6,7].

### 2.1. Driver Mutations for PV

The key driver gene in the *BCR-ABL1*-negative MPNs PV, ET and PMF is *JAK2*, encoding the tyrosine kinase Janus kinase 2, a critical mediator in erythropoiesis [8,9,10]. The point mutation c.1849G>T, affecting exon 14, causes a valine to phenylalanine substitution at position 617 in the JH2 domain.

The *JAK2*V617F variant is the most frequent mutation in *BCR-ABL1*-negative MPN, detected in 97% of PV [11], 50–60% of ET, and 55–60% of PMF cases [12]. Unlike the MPNs ET and PMF, mutations in *MPL* and *CALR* are not found in patients with PV. However, one study reported two *JAK2*V617F-negative PV patients who harboured the *CALR* type 1 (52-bp del) mutation in peripheral granulocytes at diagnosis [13]. Both patients had haemoglobin above the threshold for diagnosis (including according to the WHO’s 2016 revised criteria), moderately elevated platelet counts and normal leukocyte counts, and bone marrow biopsy showed hypercellularity. No mutation was found in *JAK2* exons 12, 13, and 14 or in *MPL* exon 10 for either patient. Nevertheless, this is only an isolated report, and subsequent studies, including the retrospective molecular diagnosis of 524 *JAK2*V617F-negative patients with suspected MPN, have failed to identify *CALR* mutations in patients with PV [14].

If the molecular analysis of *JAK2*V617F is negative, analysis for mutations in exon 12, such as N542-E543del, E543-D544del and K539L, is indicated [15] since mutations in exon 12 of *JAK2* are responsible for the majority of the remaining *JAK2*V617F-negative PV patients. These exon 12 mutations consist of deletions, insertions, duplications and substitutions of bases that affect the region linking the SH2 and JH2 domains and lead to the constitutive activation of the kinase [16]. Nevertheless, a study by Skoda and colleagues found that *JAK2*V617F and other exon 12 mutations can coexist in two separate clones in some patients [17].

Interestingly, some studies (including those performed using mouse models) have suggested that mutations in exon 12 of *JAK2* may confer a different clinical phenotype to *JAK2*V617F. For example, patients with exon 12 mutations were younger and frequently presented with erythrocytosis [18,19,20]. Nevertheless, in a European collaborative study, 106 patients harbouring the most frequent exon 12 mutations were reported to have similar rates of survival and constitutive symptoms to patients with *JAK2*V617F [16].

### 2.2. Techniques for Detection of the JAK2V617F Variant

According to the European LeukemiaNet (ELN), the standardized technique for the detection of the *JAK2*V617F variant is based on allele-specific PCR, which also allows for the quantification of the allelic frequency [21,22]. The technique of digital PCR (dPCR) is increasingly being employed since it permits the absolute quantification of the *JAK2*V617F variant without the need for the prior use of a standard curve [23,24]. For the detection of other *JAK2* variants, Sanger sequencing of genomic DNA is often employed, although it can miss mutations with allele frequencies less than 10–20% [15].

In recent years, more sensitive detection methods, such as sequencing of plasma RNA [25] and NGS [26,27], are being increasingly employed for the molecular diagnosis of patients with PV. NGS provides the advantage of potentially detecting all *JAK2* variants, including rare and/or previously undescribed mutations [26].

The most recently developed technique for the genetic profiling of patients with MPN and other haematological malignancies is the isolation of cell-free circulating DNA (ccfDNA) from patient plasma (also commonly known as liquid biopsy) coupled with its analysis by NGS [28]. A recent study assessed the feasibility of mutational analysis by the NGS of 107 patients with MPN (including 33 patients with PV) comparing the use of ccfDNA from plasma versus genomic DNA isolated from granulocytes. The authors confirmed that the sensitivity and accuracy were similar for both types of genetic material tested, with a similar mutational profile identified. However, the median VAF detected was significantly higher in ccfDNA than in DNA extracted from granulocytes (29% versus 25%, respectively) [29].

Nevertheless, despite the use of more sensitive techniques, 2–3% of patients with PV are estimated to be *JAK2* wild type. Approximately 2% of such *JAK2*-negative PV patients harbour a mutation in the gene *SH2B3* (also known as *LNK*), which encodes an inhibitor of cytokine-dependent cell growth in hematopoietic cells. Thus, loss of function mutations in *SH2B3* causes the sustained activation of JAK2 resulting in erythrocytosis [30,31]. Indeed, Lnk−/− mice models developed an MPN-like phenotype, with abnormal megakaryopoiesis and erythropoiesis, cytokine hypersensitivity, splenomegaly, fibrosis, and extramedullary haematopoiesis [32,33]. The search for mutations in *SH2B3* in *JAK2*-negative patients suspected of having PV is recommended [34], particularly in the hotspot in exon 2, although mutations in *SH2B3* have also been described in PV patients with concomitant *JAK2*V617F [35].

**Figure 1:**
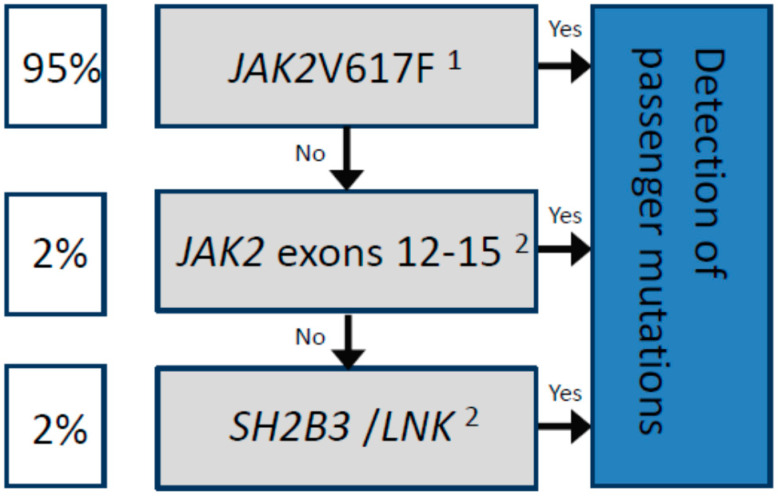
Sequential molecular analysis for the diagnosis of patients with polycythaemia vera.

In summary, when considering the molecular analysis of patients with a suspicion of PV, it is important that (i) techniques with a sensitivity of at least 5% are employed to search for driver mutations, and (ii) such testing is not simply limited to the detection of the *JAK2*V617F variant or other exon 12 mutations. For *JAK2*V617F-negative PV patients, additional mutational screening is justified, first in other exons followed by the *SH2B3* gene (Figure 1), despite the additional time and financial costs involved [34].

Driver mutations are represented in the gray boxes. The estimated frequency among patients diagnosed with PV is shown in the white boxes. In all cases, the concomitant detection of passenger mutations by NGS is recommended (blue box) for a more accurate prognosis, for example, to predict transformation to MF or AML, as well as to detect mutations associated with increased thrombotic risk, such as *TET2*. ^1^The standardized technique according to the European LeukemiaNet (ELN) is based on allele-specific PCR, which allows for the detection of the *JAK2*V617F mutation as well as the quantification of the allelic frequency [21]. ^2^The use of next-generation sequencing (NGS) is highly recommended, or other molecular techniques with a sensitivity of at least 5%.

### 2.3. Biological Impact of JAK2 Driver Mutations

The JAK2 kinase interacts with the erythropoietin (Epo), thrombopoietin (Tpo), and granulocyte colony-stimulating factor (G-CSF) receptors to regulate the normal proliferation, differentiation, and survival of myeloid cells [36]. The *JAK2*V617F driver mutation and other gain-of-function *JAK2* variants that cause the constitutive activation of the JAK2 kinase induce the proliferation of hematopoietic stem cells and provide them with a survival advantage [37,38,39]. The aberrant activation of JAK2 leads to signalling via the erythropoietin (Epo) receptor without cytokine stimulation [10,38,39] and the overactivation of several downstream signalling pathways, including JAK2/STAT5, RAS/MAPK/ERK and PI3K pathways [40]. In addition to STAT5, STAT3 is also activated in MPNs, with the phosphorylation of STAT3 and STAT5 shown to be uniformly increased in PV, in contrast to ET and PMF.

The *JAK2*V617F mutation has been shown to be clonal [10,38,39] and present in haematopoietic stem cells (HSCs) [40]. Several studies have demonstrated that the *JAK2*V617F mutant promotes genomic instability via two mechanisms: increasing levels of DNA damage and decreasing DNA damage-induced cell death. For example, aberrant JAK2/STAT5 and PI3K signalling lead to an accumulation of DNA damage via the production of high levels of reactive oxygen species and the stimulation of homologous recombination, leading to increased amounts of double-strand breaks [41,42,43,44] that in turn impede replication fork progression [45].

Indeed, Quelle et al. linked the misregulation of the JAK2 signalling pathway to the suppression of p53-mediated cell death in response to DNA damage in hematopoietic cells as early as 1998 [37]. JAK2/STAT5 activation promotes the G1-S phase transition of the cell cycle due to the increased degradation of p53 as a result of enhanced MDM2 translation [42,46]. Impairment of the intra-S checkpoint response in erythroblasts from PV but not ET patients was also shown to result in increased DNA damage due to attenuated p53 signalling [45]. Furthermore, JAK2/STAT5 pathway activation induces overexpression of the STAT5 target gene *BCL2L1*, which codes for the anti-apoptotic protein Bcl-xl [37,47], as well as inhibiting its deamidation [48] preventing apoptosis in response to the detection of DNA damage. Together, these mechanisms result in an accumulation of proliferating malignant cells with unresolved DNA damage.

Interestingly, treatment of mice engrafted with *JAK2*V617F-mutated bone marrow cells with the antioxidant N-acetylcysteine resulted in reduced levels of DNA damage and decreased splenomegaly [44], while the dietary intervention of patients with MPN, including N-acetylcysteine supplementation, led to a certain improvement in symptom burden [48,49]. These results suggest that the reduction of damaging ROS effects with antioxidants may potentially be a promising therapeutic approach.

The principal model of pathogenesis of PV and other MPN is based on genomic instability; however, increasing evidence is also supporting the consideration that the clonal evolution characteristic of MPN and other cancers may be triggered by chronic inflammation and an escape from anti-tumour immunological surveillance [50,51]. Indeed, the incidence of second cancer has been reported in up to 10% of all MPN patients [52,53], with a second solid or lymphoid tumour detected in 31 of the 1042 PV patients (3.0%) analyzed from the ECLAP study [54].

Additionally, smoking has been shown to be a significant risk factor for MPN development due in part to the persistent stimulation of myeloid cells [55], with a hazard ratio for daily smokers of 2.5 for any MPN, and of 4.3, 1.8 and 1.7 for MF, ET and PV, respectively [56].

Whether immune dysregulation can be considered an MPN disease “driver” or not remains to be fully elucidated [57], but the connection between chronic inflammation and vascular function, and atherosclerosis in particular, is indisputable [58,59]. For a comprehensive recent review on the role of inflammation in MPN, we refer readers to the article by Di Battista and colleagues published in this special edition [60].

### 2.4. Molecular Differentiation of PV from Other Haematological Neoplasms

Presence of the *JAK2*V617F variant, although a distinguishing feature of PV, is not a specific molecular marker for PV. Nor is it a marker for the *BCR-ABL*-negative MPN, having been detected in other haematological neoplasms including AML, myelodysplastic syndrome, and chronic myelomonocytic leukaemia [61,62]. The variant has even been found at low levels in the peripheral blood of healthy individuals [63].

One of the first molecular markers identified for the diagnosis of patients with PV was the polycythemia rubra vera 1 gene (*PRV1*). Granulocytes isolated from the peripheral blood of patients with PV and some cases of ET had an increased expression of *PRV1* together with a decreased expression of *MPL*, with a positive correlation observed between *PRV1* expression and splenomegaly, helping to differentiate MPN from secondary erythrocytosis and thrombocytosis [64,65]. However, the use of *PRV1* lost popularity when the *JAK2*V617F driver mutation was discovered in 2005 [10] and *PRV1* was not included as a diagnostic marker for PV in the 2008 WHO classification [1]. Moreover, *PRV1* expression does not discriminate PV from other *BCR-ABL1*-negative MPN [66].

A second molecular marker whose expression in circulating mononuclear cells was shown to be significantly increased in 87% of patients with PV but not secondary polycythaemia is the insulin-like growth factor 1 receptor (IGF-1R) [67,68]. The levels of *IGF1R* in PV were significantly different in patients with PV compared with patients with ET or PMF, although expression of *IGF1R* was significantly higher in patients with the other MPN compared to controls [68].

Distinguishing PV from ET can be complicated due to the shared phenotypic characteristics of the two diseases, particularly the differential diagnosis of PV from *JAK2*V617F-positive ET. Prior to the 2016 WHO update to the diagnostic criteria for MPN (in which haemoglobin and haematocrit diagnostic thresholds for PV were lowered to 16.5 g/dL and 49% for men, and 16 g/dL and 48% for women, respectively, and bone marrow biopsy was made mandatory, except for cases fulfilling the 2008 WHO diagnostic criteria [2]), PV patients with thrombocytosis at disease presentation were frequently misclassified as *JAK2*V617F-positive ET patients [69,70,71,72]. It was shown that “masked PV” [71,72] could be distinguished from ET based on red blood cell mass (RCM), with masked PV presenting an increased RCM above 125% [70,73].

This differentiation is important for the prognosis of a patient with MPN, since a higher rate of thrombosis exists among patients with PV than ET, as well as a higher rate of transformation to secondary myelofibrosis (SMF) and AML in PV than ET [71,74]. Importantly, the combined rate of transformation to SMF and/or AML for masked PV was 1.60 per 100 patient years compared to 0.97 for overt PV [74]. These inferior outcomes may possibly be due to a more aggressive form of the disease, the lower intensity of treatment for masked PV patients misdiagnosed with ET, or perhaps due to a later diagnosis [75]. For example, masked PV patients misclassified as *JAK2*V617F-positive ET would not be phlebotomized. Moreover, misdiagnosed younger patients without prior thrombosis would be categorized as low risk (according to current international guidelines for ET patients) and thus would not even receive cytoreductive treatment, even in those presenting with leucocytosis and thrombocytosis [75].

The differentiation between PV and ET at a molecular level can also be complicated by the presence of passenger mutations in genes common to both MPN. However, mutations in the genes *KMT2A* (*MLL*) and *TP53* were more common in patients with PV (13.64% and 6.25%, respectively) than patients with PMF (3.45% and 0%, respectively) or ET (0% and 0%, respectively) [76]. Some studies have characterized the expression of several genes and proteins in an attempt to differentiate PV. For example, the overexpression of *NFE2* was described in patients with PV and not in patients with ET [77], while high expression of the proteins HSP70 and calreticulin in the membrane of erythrocytes of patients with PV compared with patients with ET suggested an important role for these factors in the proliferation and erythroid function of PV, respectively [78,79]. Moreover, the expression of microRNAs (miRNA), single-stranded non-coding RNA 18–24 nucleotides in length that regulate gene expression by binding to target mRNA, was shown to be different between patients with PV and those with ET (Table 1), as well as between patients with PV and control patients [80,81]. For example, miR-145 and miR-451 were upregulated in nucleated erythroid cells cultured from progenitor cells from patients with PV compared to patients with ET, whereas miR-575 was upregulated and miR-196b was downregulated in PV patients compared to healthy controls [81].

Nevertheless, the JAK2 allelic burden is sometimes employed to differentiate patients with PV from patients with ET [82,83,84]. Homozygosity of JAK2V617F (represented by a VAF of 50% or more) or a high JAK2V617F allele burden resulting from mitotic recombination of chromosome 9 or 9p loss of heterozygosity [10,39] occurs more frequently in patients with PV while it is significantly less common in patients with ET [83,85]. This observation was confirmed in transgenic mice, in which the increased expression of JAK2V617F resulted in a change of phenotype from ET to PV [86,87].

### 2.5. Germline Predisposition for Developing PV

A hereditary basis of PV (and other MPNs) was first suggested by observations of a particularly high incidence of PV among people of an Ashkenazi Jewish descent [88], although the common genetic predisposing factor among this population has still yet to be identified. However, the familial basis of PV may be more frequent than previously anticipated, with a population study finding a 5.7-fold increased risk of PV among the first-degree relatives of patients with MPN [89]. Moreover, an interview-based investigation of family history identified familial cases of MPN in 18 of 206 patients with PV (8.7%), with 13 families having first-degree relatives affected by PV [90].

Genetic studies observed that carriers of the *JAK2* 46/1 haplotype (GGCC) had a higher susceptibility to develop MPN [91,92]. Specifically, carriers of the 46/1 haplotype had a 3.7-fold increased risk of acquiring the *JAK2*V617F mutation [93], a higher susceptibility to acquire a *JAK2* exon 12 mutation [19,94], and an increased disposition (odds ratio of 2.3) for the development of PV versus ET [85].

However, despite the predominance of the *JAK2*V617F mutation among patients with PV, earlier studies determined that this “driver” mutation was not always disease initiating [95] and might occur as a later secondary genetic event in some patients with MPN [96,97]. Familial cases of MPN, where different family members have a different driver mutation and/or a different MPN, support a pre-*JAK2* event and are suggestive of an unknown germline event that predisposes to MPN (unpublished observations, [98]).

One hypothesis is that *JAK2* germline mutations, such as *JAK2*T108A and *JAK2*L393V, are the genetic predisposing factor that precedes the acquisition of *JAK2*V617F in MPN [99]. Albeit of uncertain significance, these germline mutations were identified in PV patients as co-existing predisposing mutations with somatic *JAK2*V617F, and have also been reported in an adenocarcinoma cell line and in diffuse large B cell tumours, respectively, supporting the observation that they provide a proliferative advantage. Nevertheless, their effect on disease initiation has not been fully determined.

Other predisposing germline mutations have been identified among patients with familial MPN, including mutations in the *RBBP6* gene, encoding an E3 ubiquitin ligase known to promote the degradation of p53. Variants such as *RBBP6*R1569H were reported to lie in the p53-binding domain, and thus are predicted to affect p53 function [100]. Other germline predisposition variants for MPN include the single nucleotide polymorphism (SNP) rs2736100 in the gene encoding the telomerase reverse transcriptase *TERT* [101]. In fact, both the *TERT* SNP rs2736100 and the *JAK2* GGCC haplotype had an additive effect on MPN risk in the presence of an *RBBP6* mutation and a higher VAF [102].

Genome-wide association studies (GWAS) have confirmed that various loci associated with telomere length, including rs7705526 and rs2853677, are predisposing factors for MPN risk [103]. Specifically, an increased risk of MPN development was observed for individuals with longer telomere length measured in leukocyte (LTL), as determined using a combined “teloscore” from 11 telomere-length-associated SNPs [104]. The authors of the study concluded that longer telomeres are a risk marker for MPN development.

Nevertheless, a ground-breaking study, presented at this year’s American Society of Hematology (ASH) Annual meeting, demonstrated that driver mutations, such as *JAK2*V617F were frequently acquired in childhood or even in utero, many decades before an MPN diagnosis [105]. The authors observed a mean time of 34 years between driver mutation acquisition and MPN presentation in patients where *JAK2*V617F was the only or the first driver mutation. In 4 of the 10 patients analyzed, *DNMT3A* single-nucleotide variants (SNVs) were the driver event, followed by the acquisition of *JAK2*V617F, although the *DNMT3A* mutations were also acquired in childhood or in utero. The authors concluded that the observed latency between driver mutation acquisition and MPN presentation suggests that other factors, including germline, must impact the selection of clones harbouring driver mutations and favor their clonal expansion. Indeed, while normal hematopoietic stem cells accumulated approximately 18 somatic mutations per year, clones harbouring driver mutations such as *JAK2*V617F accumulated 1.5–5.5-fold more mutations per year and were also found to have shorter telomeres.

### 2.6. Passenger Mutations

Genetic analyses have led to the description of diverse non-driver mutations in patients with PV, particularly as a result of NGS studies. These include SNVs in genes that encode proteins that regulate epigenetic processes, such as *TET2, DNMT3A, ASXL1, EZH2*, and *IDH1/2*, as well as proteins implicated in the splicing of messenger RNA, such as *SRSF2, SF3B1* and *U2AF1* [12,106], with non-driver mutations detected by NGS in 53% and 75% of PV patients according to two recent studies [107,108].

The effect of passenger mutations on the prognosis of the disease is still not well characterized, although some studies have associated SNVs in *ASXL1, SRSF2*, and *IDH1/2* with poor survival [107,109,110]. Mutations in two novel genes were reported at this year’s ASH Annual meeting to have prognostic value in MPN. SNVs in the gene *NFE2* (nuclear factor erythroid 2), with a role in the maturation and differentiation of erythroid cells, were found to be independently associated with shortened time to blastic transformation (with a hazard ratio of 9.9) and overall survival. Such mutations were most common in PV patients compared to PMF and ET, detected in 7.3%, 5.3% and 3.6%, respectively [111]. Similarly, SNVs in the gene *STK11* (serine/threonine kinase 11), encoding a kinase with tumour suppressor function, were found to promote leukemic progression in patients with MPN [112].

However, the clinical relevance of some passenger mutations in patients with MPN is still a matter of debate. For instance, the presence of somatic mutations in hematopoietic tissue is positively correlated with age and confers a selective advantage leading to clonal haematopoiesis of indeterminate potential (CHIP, with an established cut-off of ≥2% VAF of in peripheral blood) [85,113,114,115,116,117,118,119].

The most frequently mutated CHIP-associated genes are *TET2, DNMT3A, ASXL1* (commonly referred to as the DTA genes), and *JAK2*. Recent studies have found an association between the accumulation of DTA SNVs and a higher risk for developing myeloid neoplasms, including *BCR-ABL1-*negative MPN and AML, as well as cardiovascular pathologies and a higher prevalence of developing derived complications from diabetes [119].

*TET2* SNVs are particularly common in PV, detected in between approximately 10% and 36% of patients [12,106,108,120,121], and are frequent in both *JAK2*V617F-positive and -negative MPN. Experiments in mice suggested that mutated *TET2* confers a clonal advantage to cells mutated in *JAK2* that accelerates their proliferation [122,123]. Moreover, the acquisition of a mutation in *DNMT3A* followed by mutation in *JAK2* was shown to lead to an ET phenotype, whereas the acquisition of the *JAK2* mutation first was shown to result in a PV phenotype [124].

Nevertheless, there remains some controversy over whether the presence of CHIP mutations is sufficient for the clonal expansion that results in the emergence of MPN since DTA mutations (as well as the *JAK2*V617F mutation) are often identified in healthy individuals [116]. An alternative hypothesis is that such mutations simply predispose individuals to acquire mutations in other oncogenes or other genomic abnormalities necessary to drive clonality [105].

Sequencing studies and colony genotyping are helping to determine clonal evolution [125,126] and the order of mutation acquisition. For example, mutations including *NFE2, TP53, NRAS*, and *PPM1D* were found to be acquired significantly later in MPN disease [85]. A seminal study by Ortmann et al. investigated the influence of the order of mutations in *JAK2* and *TET2* in patients with MPN [127]. Firstly, the authors observed that among MPN patients with mutations in both genes, the prior acquisition of *TET2* mutations diminished the proliferative effect of the *JAK2*V617F mutation. Secondly, they found that PV was more common among *JAK2*-first patients compared to ET or PMF. Finally, they concluded that the order of mutation acquisition influenced the age at which a patient presented with an MPN, with *JAK2*-first patients presenting at a younger age compared with *TET2*-first MPN patients. This observation was supported by a recent study which concluded that the order of mutation acquisition was associated with time to MPN presentation [105].

## 3. Prognosis

Patients with PV have a median overall survival of 13.5 years [128]. At diagnosis, PV patients are classified into groups of high, intermediate or low risk for overall survival according to the clinical variables of patient age, leukocyte count and history of venous thromboembolic events (Table 2) [129].

Until very recently there were no well-characterized molecular determinants of prognosis that could be used in the clinic to predict the survival of patients with PV [7], with the ELN score only considering patient age and history of thrombosis [21]. However, the recently proposed Mutation-enhanced International Prognostic Scoring System (MIPSS-PV) includes the mutation *SRSF2* due to its association with worse overall survival (Table 3) [130] to classify patients into low-risk (score of 0–1), intermediate-risk (score of 2–3) or high-risk (score ≥ 4) groups.

### 3.1. JAK2V617F Allelic Burden

Although quantification of the *JAK2*V617F allelic burden is not obligatory for the diagnosis and follow-up of patients with PV according to the WHO criteria [1,2], patients homozygous for *JAK2*V617F were shown to be more symptomatic than patients who were heterozygous. Specifically, a positive correlation was described between a high allelic frequency of mutated *JAK2* and clinical presentation including pruritus [131,132,133], myelopoiesis [11,132], splenomegaly [11,131,132,134,135], and a negative correlation with the platelet count [11,132,135,136].

Moreover, determination of the *JAK2*V617F VAF at diagnosis can provide important prognostic information. For example, in PV, a higher mutant allele burden has been associated with an increased incidence of progression to SMF [11,83,131,137].

Both of these observations are suggestive of a “dosage” effect (reviewed in [98]), where a high *JAK2*V617F burden results in a stronger activation of JAK2/STAT (and possibly other) signalling pathways, leading to the stronger activation of downstream genes [138], and thus a more aggressive phenotype. In support of this hypothesis, the expression level of several JAK2 target genes—such as *PRV1* [138] and *NFE2* [139]—was shown to be dose dependent for the mutant allele burden.

A high *JAK2*V617F allele burden in patients with PV has also been associated with a risk of thrombotic events [140,141,142]. A study of 1306 patients with MPN including 397 patients with PV, observed an incidence of thrombosis at a diagnosis of 21% for homozygous and 15% for heterozygous *JAK2*V617F [142], with *JAK2*V617F homozygosity at diagnosis identified as an independent risk factor for major vascular events in a multivariate analysis [141]. A second study reported a 7.1-fold increased risk of major vascular events for patients with a mutant allele of 75% and above compared to less than 25% VAF during follow-up [130]. However, one study associated a significantly increased risk of venous thromboembolism with a much lower mutant allele burden, of just 20% or more [131]. Despite the differences in the VAF threshold reported to have a negative effect on thrombotic risk, it is clear that patients with a higher *JAK2* mutant burden are at risk of both vascular events and fibrotic transformation.

Nevertheless, one study’s findings contradict the prognostic impact of the *JAK2*V617F allelic burden in patients with PV. Gene expression profiling of CD34+ cells isolated from 19 patients with PV led to the identification of a group of patients with a more aggressive disease—with higher rates of blastic transformation and worse overall survival—and a group with a less aggressive form despite similar *JAK2*V617F allele frequencies in both groups. Patients in the group with more aggressive PV showed differential expression of the NOTCH and SHH pathways as well as inflammatory cytokines and histone genes [143]. Interestingly, this study also revealed gender-specific differences in the expression profile in that men with PV had significantly more differentially regulated genes than women with PV.

### 3.2. Cytogenetics

Cytogenetic abnormalities are detected in approximately 10–20% of PV patients at diagnosis, with some of the most common alterations including gain of chromosomes 8 and 9, and deletion of (1p), (13q) and (20q) [144,145,146].

Although some studies have not shown a prognostic difference based on cytogenetic characteristics [146], several groups have reported, as association between a higher risk of fibrotic progression and the presence of chromosome 12 abnormalities [147], the gain of (1q) [148], and an abnormal karyotype. Moreover, the majority of investigations, including one by the International Working Group for Neoplasms Research and Treatment (IWG-MRT), have reported an association between an abnormal karyotype and transformation to myelodysplastic syndrome or AML [146,148,149,150] and poorer overall survival [145,148,150].

Interestingly, a retrospective study of 422 PV patients with cytogenetic information available at diagnosis observed dynamic changes associated with progression to AML, including an increased frequency of abnormal karyotype, increasing from 20% to 90% in the blast phase, as well as a different distribution of cytogenetic abnormalities [149]. For example, complex karyotype as well as deletion of (5q)/chromosome 5, 7q/chromosome 7, and (17p)/chromosome 17/i(17q) were more common in the blast phase, whereas the gain of chromosomes 8 and 9 were more common in the chronic phase of the disease. Using this information, the authors stratified patients into low (normal karyotype; +8, +9; or presence of one other alteration), intermediate (del(20q), +(1q) or presence of two other alterations) and high-risk (complex karyotype) groups according to their cytogenetics at diagnosis [148].

### 3.3. Thrombosis

The *BCR-ABL*-negative MPNs PV, ET and PMF are associated with a high frequency of haemorrhages and thrombosis, including myocardial infarction, ischemic stroke, deep vein thrombosis, and thrombo- and pulmonary embolisms. Notably, vascular events are the major cause of morbimortality among patients with PV, with the incidence of events estimated between 6% and 17% over three years [151,152].

Thrombotic risk prediction tools have been developed for each *BCR-ABL1*-negative MPN subtype. However, thrombotic events are frequent among patients with PV, even among those classified as low-risk according to the current ELN recommendations [21,153], as well as patients who receive cytoreductive or anti-aggregant treatment [154].

Several common thrombophilia markers are associated with an increased risk of developing vascular events in patients with PV (reviewed in [155]), including high levels of C-reactive protein in blood serum and SNVs in the factor V-Leiden (*F5*) gene [156,157,158]. Higher factor VIII levels, a known risk factor for venous thrombosis and coronary artery disease [159], were identified in PV patients compared to healthy subjects, with levels of 141 versus 98 IU/dL, respectively [160]. The PV patients were also found to have significantly higher VWF antigen and activity which, by multivariate analysis, was predicted for by *JAK2*V617F allelic burden. Nevertheless, the ELN does not currently recommend thrombophilia testing as part of routine clinical practice in PV or MPN patients [21,153].

Other prognostic biomarkers reported for PV include an abnormal karyotype, identified as a risk factor for venous thrombosis [145], and leucocytosis, a risk factor for arterial thrombosis, although its impact on venous thrombosis remains inconclusive [161,162].

The International Prognostic Score of thrombosis in World Health Organization-essential thrombocythemia (IPSET-thrombosis) was the first prognostic algorithm to incorporate the *JAK2*V617F mutation to better predict the risk of thrombosis among ET patients [163], although the influence of *JAK2*V617F in PV patients as well as the clinical significance of the allelic burden on thrombotic risk remain to be determined. Some studies have found that a higher *JAK2*V617F allelic burden was associated with increased risk of thrombosis in PV and ET [164]. For example, *JAK2*V617F allele burden cut-offs for PV > 25.7% and > 90.4% were established for arterial thrombosis and for venous thrombosis, respectively [165].

Moreover, elevated levels of the inflammatory biomarker C-reactive protein in peripheral blood were associated with the PV phenotype (vs. essential thrombocythemia), older age, cardiovascular risk factors and a *JAK2*V617F allele burden over 50% [59,166], further supporting suggestions of a possible link between inflammation and atherosclerosis [58,59,60]. A seminal study from 2017 identified an association between mutations in the DTA and *JAK2* genes and coronary disease [119]. Mechanistically, mutations to DTA genes are suggested to alter methylation patterns and thus cause an increased transcription of pro-inflammatory genes triggering atherosclerosis [119,167,168]. To support this theory, Fuster and colleagues studied the effect of the expansion of TET2-deficient cells in atherosclerosis-prone, low-density lipoprotein receptor-deficient mice and observed that the partial reconstitution of the bone marrow with TET2-deficient cells was sufficient to generate clonal expansion, which was also associated with a marked increase in the size of atheromatous plaques and the increased secretion of pro-inflammatory cytokines such as IL-1β [167].

In accordance with these results, Jaiswal et al. found that individuals with clonal hematopoiesis had a 1.9-fold higher risk of developing coronary disease and a 4-fold higher risk of myocardial infarction, rising to 12.1-fold in individuals with the *JAK2*V617F mutation [119]. In support of the link between constitutive JAK2 activation and increased risk of vascular events, the rs3184504 SNP in the *SH2B3* (*LNK*) gene was also associated with an increased risk of coronary heart disease [169]. This variant, encoding the TT genotype (R262W), caused an increased platelet count, leukocytosis and hypertension. At the molecular level, *SH2B3* mutation increased megakaryopoiesis via the upregulation of AKT signalling, shown to be specifically upregulated in platelets and to promote prothrombotic and proatherogenic aggregates [170]. Specifically, our group recently showed that DTA mutations were associated with increased thrombotic risk in patients with PV, with a specific interaction identified for pathogenic *TET2* mutations [108].

Activated leukocytes (including neutrophils and monocytes) appear to play an important role in the development of platelet aggregates, markers of a prothrombotic state. In addition, there are reports that leuko-platelet aggregations directly correlate with platelet and leukocyte counts [171,172], adding weight to leukocytosis as a thrombotic risk factor. Interestingly, and as previously mentioned, the expression of CD177 (*PRV-1*), a GPI-linked cell surface glycoprotein with a role in neutrophil activation, was increased in PV patients, and to a lesser extent in ET and PMF patients [64,65]. Indeed, higher CD177 expression was associated with an increased risk of thrombotic and bleeding complications due to increased circulating neutrophils [173,174]. For a complete and recent review on the role of neutrophils in thrombosis, we refer readers to the article by Ferrer-Marín and colleagues published in this special edition [175]. It is clear that we need to further elucidate the molecular mechanisms of leukocyte–platelet interactions in order to design better prophylactic treatment plans for the prevention of thrombotic complications in patients with PV.

### 3.4. Molecular Indicators of Transformation

Progression to fibrotic disease (post-PV MF) or blastic disease, both associated with dismal survival rates, is estimated to occur in 10% and 15% of PV patients, respectively [3]. Molecular indicators of fibrotic progression from PV are not well characterized. Some evidence exists suggesting an association between chromosome 12 abnormalities and an increased risk of developing post-PV MF [176]. Evidence also exists to support a significantly higher *JAK2*V617F allelic frequency [11,83,131,137,176] or a progressive increase [177] in patients who transform to SMF. Unlike in PMF, the presence of additional high-risk mutations (*ASXL1, EZH2, IDH1, IDH2* or *SRSF2*, as single or multiple mutations) was not correlated with reduced leukaemia-free survival or indeed overall survival, with the exception of mutations in *SRSF2*, associated with reduced survival [178,179].

As the molecular profile of post-PV MF was shown to be considerably different from PMF [180], the Myelofibrosis Secondary to PV and ET Prognostic Model (MYSEC-PM) was specifically developed to predict survival in post-PV/post-ET MF (Table 4) [181].

The score was shown to more effectively assess prognosis in post-PV MF patients, with poor agreement for risk classification between the DIPSS and MYSEC-PM scores when applied to the same post-PV patients [180,182]. Therefore, the MYSEC-PM is the most appropriate prognostic score for post-PV MF patients rather than the International Prognostic Scoring System (IPSS) or Dynamic IPSS (DIPSS) scores, both of which were developed for PMF.

Interestingly, in a retrospective study of 376 patients with SMF, including 188 post-PV MF, monosomal karyotype (observed in 8.5% of the SMF patients studied) was associated with significantly worse survival, with a median of 2 years, independently of the MYSEC-PM risk group [183].

In terms of blastic transformation, it is also clear that the genetic profile of patients with post-PV AML differs from that of *de novo* AML. For example, mutations in *FLT3* and *NPM1* are very uncommon and the *JAK2*V617F-positive clone is frequently lost in transformation to AML [184,185]. Moreover, abnormal karyotype is a risk factor for leukemic transformation in PV and ET [145,146,147,150], estimated to occur in 80% of PV cases that progress [146].

In some cases, the transformation from PV to AML is associated with the loss of function of TP53 due to an acquired mutation in the *TP53* gene leading to clonal dominance [85,177,186,187,188]. In fact, in a mouse model, the expression of *JAK2*V617F in combination with the loss of TP53 was demonstrated to cause an AML phenotype [188]. This result was reflected in a later NGS study, which observed that post-PV/-ET AML patients with a *TP53* mutation had a survival rate at 12 months of just 18% compared to 48% for patients without a *TP53* mutation [187].

Other mutations associated with a higher risk of leukemic transformation include *ASXL1, EZH2, RUNX1* and *SRSF2* [85,130,187,189]. Indeed, a recent French study that investigated the time between diagnosis and leukemic progression in 49 patients with post-PV/ET AML (including 24 post-PV) observed that mutations in *IDH1/2, RUNX1*, and *U2AF1* were associated with a shorter time to transformation, while mutations in the genes *TP53, NRAS*, and *BCORL1* were associated with a longer time to transformation [187]. Such mutations are often detectable at low allelic frequencies in earlier stages of the disease, while others may appear later in the progression [186,187,188].

*TET2* was also observed in some studies to be a gene whose mutation is associated with the leukemic transformation of PV patients. One study that investigated mutations in 63 patients with AML secondary to an MPN found that mutations in *TET2* were acquired at transformation in 43% of cases [190], and the acquisition of mutations in *TET2* in patients with PV *JAK2*V617F was associated with transformation to AML and reduced survival [120]. Nevertheless, the prognostic impact of *TET2* mutations, and indeed other mutations associated with CHIP, is not entirely clear. For example, when the impact of additional mutations on the risk of leukemic transformation was investigated, the presence of >1 additional SNV was associated with a higher risk of leukemic transformation and this association was even higher when *TET2* SNVs were removed [187]. Moreover, mutations in *ASXL1* were observed at similar allelic frequencies before and after transformation, suggesting that they may not contribute to the dominance of the emerging leukemic clone [188].

One of the largest sequencing studies performed to date, on 2035 MPN patients (including 356 PV patients), identified eight genetic subgroups [85]. The authors developed a sophisticated prognostic model based on 63 clinical and genomic variables to estimate a patient’s probability of leukemic transformation. These findings have now been adapted into a personalized risk calculator, available online (https://cancer.sanger.ac.uk/mpn-multistage/, accessed on 26 November 2020).

## 4. Treatment

Thrombosis is the most common cause of morbidity and death, followed by the complications of myelofibrosis and development of leukaemia. As such, the primary treatment goals in MPN are to avoid thrombosis and bleeding and minimize transformation to myelofibrosis or AML while treating MPN-related symptoms to improve the quality of life of patients with PV [6,7,191].

Frontline strategies to prevent vascular events include prophylactic aspirin with phlebotomies as and when required to maintain haematocrit (HCT) levels below 45%. In addition, patients often require cytoreductive treatment, most commonly hydroxyurea (HU), according to recent European guidelines [6,7,21,153]. However, approximately 15–20% of patients develop intolerance or resistance to HU with continued use [192,193].

To date, there is very little information on how to identify patients with PV at risk of developing resistance to HU treatment. However, one recent study used NGS to molecularly characterize 37 PV patients with resistance or intolerance to HU [194]. The authors found that patients with a homozygous *JAK2* mutation had a higher risk of developing resistance after 5 years of HU treatment compared to those with a heterozygous *JAK2* mutation (27% vs. 14.5%), while for those with a *TP53* alteration (mutation or copy number alteration) the probability increased to 64% [194].

For HU-resistant or -intolerant patients, ruxolitinib is the only JAK2 inhibitor currently approved by the European Medicines Agency (EMA) and the Food and Drug Administration (FDA) as second-line treatment [195,196], following the positive results of the clinical trials RESPONSE, in PV patients with splenomegaly (NCT01243944, ClinicalTrials.gov) [197], and RESPONSE-2, in PV patients without splenomegaly [198]. Disappointingly, a phase 2 study with momelotinib, another JAK2 inhibitor, in patients with PV (NCT019898828, ClinicalTrials.gov) was terminated early due to low efficacy [199].

One particularly positive observation from clinical trials with ruxolitinib was the reduction in the incidence of thromboembolic events, 5-fold lower in the group of patients receiving ruxolitinib in comparison with the group receiving the best available treatment (BAT; HU in the majority of patients) in Week 32 of treatment, and 4.5-fold lower in Week 80, although this was not a primary study objective [195,196]. These observations were confirmed by a meta-analysis which included data from 750 PV and MF patients, including those who participated in the RESPONSE trial [200]. In addition, the highly anticipated results of the MAJIC clinical trial (EudraCT 201100527918) should provide more information on the frequency of thrombotic events in patients with PV treated with ruxolitinib.

Also noteworthy is the recent European Medicine Agency (EMA) approval of Ropeginterferon alpha-2b (Besremi^®^) for the treatment of adult patients with polycythaemia vera [201] as a result of the PROUD, CONTINUATION-PV [202] and Low-PV clinical trials [203]. Besremi^®^ can be used as first-line treatment but may also offer an alternative for HU- and/or ruxolitinib-resistant or -intolerant patients.

Ropeginterferon alfa-2b is a monopegylated interferon (IFN) that can be administered once every 2 weeks. The molecule binds to interferon α/β receptors (IFNAR) of endogenous interferon cytokines on the cellular membrane of many blood cells, including B cells, NK cells, mononuclear cells and macrophages, as well as endothelial cells, epithelial cells, hepatocytes and many other cell types [204]. Although the mechanism of action of IFN remains to be elucidated, it does have pro-apoptotic properties, activating the expression of caspase and tumour necrosis factor-related apoptosis-inducing ligand (TRAIL) [205].

**Figure 2:**
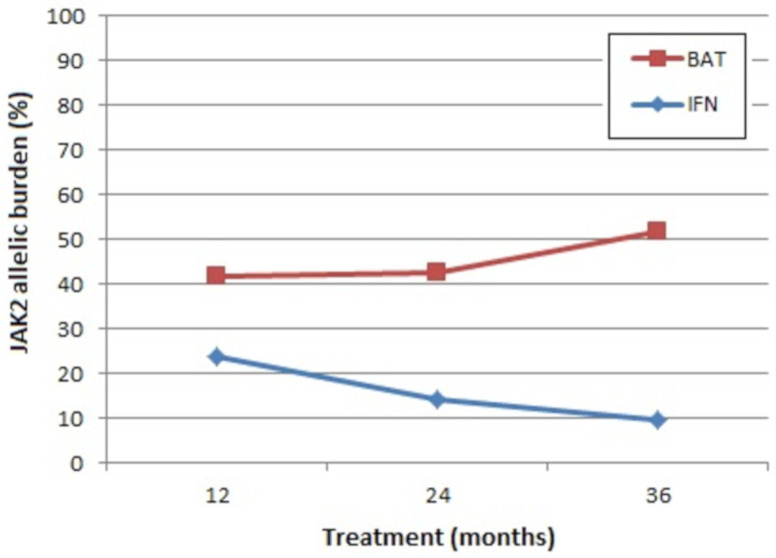
Mean absolute levels of *JAK2* allelic burden. BAT: best available therapy (100% of the patients received hydroxyurea at least once), IFN: pegylated interferon-alpha 2b. Representation of results from CONTINUATION-PV (adapted from [201]).

Advantages of IFN include that it is not leukomogenic in comparison to HU [201,206], and its long-term use (>18 months) leads to a sustained decrease in *JAK2* allelic burden in the majority of patients (Figure 2) [201]. Nevertheless, the clinical relevance of this reduction remains to be determined [201]. For example, is the reduction in *JAK2* allelic burden reflected in the patient’s response to therapy with a reduction in symptom burden? A lower incidence of fibrotic or leukemic transformation? Improved overall survival?

Other areas of doubt in relation to treatment with IFN include its effect on thrombosis. One recent study observed the significant elevation of the pro-coagulant biomarkers thrombin, fibrinogen, Von Willebrand factor, shear-induced platelet aggregation, and factor VIII coagulant activity (FVIII:C) in the blood plasma of patients with PV or ET treated with IFN compared to those treated with HU or no treatment, independently of gender and patient age [207]. Whether these reported effects of IFN treatment on the haemostatic profile of PV patients are associated with an increased thrombotic risk remains to be determined; however, important differences were reported between the risk of thromboembolic events for PV patients treated with IFN (8.7%, 11/127) versus best-available therapy (BAT, 5.5%, 7/127) [201].

The approval of Besremi^®^ is the first new treatment option for patients with PV in many years, although several others are in different stages of development and have shown promising preliminary results, such as the histone deacetylase inhibitor givinostat and the hepcidin-mimetic PTG-300. Treatment strategies are outside the scope of this review on the molecular-informed diagnosis and prognosis of patients with PV; thus, we refer readers to a comprehensive review of current treatment options as well as novel agents in a recent volume of *International Journal of Molecular Sciences* [208].

## 5. Future Perspectives

There is no doubt that the application of NGS in haematology clinics is helping to confirm the diagnosis of patients with PV by detecting rare mutations in *JAK2* or *SH2B3*, as well as identifying germline mutations. A greater understanding of the genetic basis of MPN might mean that the screening of family members is feasible in the future.

In the prognosis of patients with PV, the incorporation of molecular markers into prognostic algorithms such as the MIPSS-PV and MYSEC-PM is helping aid more precise risk stratification. Cytogenetics have yet to be included in any risk prediction model, yet karyotype has prognostic value. Since vascular events remain the major cause of morbidity and mortality for PV patients, the identification of biomarkers associated with thrombotic risk and their incorporation into current algorithms to predict vascular events constitute an important area of ongoing investigation.

Other gray areas remain, including the impact of the allelic burden of the most common variant in PV patients, *JAK2*V617F, on patient outcomes. Moreover, the application of NGS has failed to improve the prediction of disease transformation. Continued molecular studies are required to improve our understanding of the molecular basis of fibrotic and leukemic transformation to identify biomarkers for patient risk stratification. In addition, the order of mutation acquisition and its contribution to clonal expansion and leukemic transformation are matters that remain to be fully elucidated.

The outcome of PV patients who do progress remains dismal. Many other mutations and molecular mechanisms necessary for leukemic transformation are probably still yet to be identified. Thus, there is hope that continued molecular analyses will lead to the recognition of pathways that could be targeted for the development of novel therapeutic strategies to prevent transformation.

To date, the only curative option for PV patients who transform is a hematopoietic stem cell transplant (HSCT). It remains to be determined whether somatic mutations can predict outcome after the allogeneic HSCT of PV patients as observed for *CALR, IDH2*, and *ASXL1* mutations in patients with myelofibrosis [209].

NGS studies are also providing molecular information that is guiding therapy, such as the molecular characterization of PV patients at risk of developing HU resistance [196].

Although recently approved by the EMA, many doubts remain around the use of IFN. Should only high-risk PV patients be treated with first-line IFN or low-risk patients too? The impact of IFN on the incidence of thrombosis and transformation also remains to be elucidated. Moreover, studies identifying molecular indicators of patients who respond well to IFN treatment are required. Finally, the effect of acquired passenger mutations on molecular resistance to IFN will be an interesting future area of research. Preliminary observations showed that patients who failed to achieve a complete molecular response had a higher frequency of mutations in *TET2, ASXL1, EZH2, DNMT3A*, and *IDH1/2* genes prior to treatment but also acquired more mutations during IFN therapy [210]. Nevertheless, the study analyzed patients treated with Ropeginterferon alfa-2a rather than the -2b form approved by the EMA.

Finally, as a result of the continual advancements in our understanding of the impact of somatic mutations and the complex interactions between them, although the presence of a certain mutation may not currently be prognostic, a targeted therapy may potentially be developed that may allow us to optimize treatment for a patient in the future. Therefore, it is essential that patients’ genetic material is stored in biobanks.

## 6. Conclusions

Advances in molecular techniques, particularly NGS technologies, have helped improve our understanding of the molecular basis of PV. As a result of this knowledge, molecular testing is being increasingly applied in haematology laboratories to inform the diagnosis of patients with PV, and its routine use in clinical practice should be encouraged. Moreover, we believe that molecular information from genetic studies should be integrated into clinical decision models to refine risk stratification and so improve patient management via the individualization of treatment strategies.

The impact of *JAK2*V617F allelic burden and the presence of passenger mutations in the development of thromboembolic events, leukemic progression and overall survival have yet to be convincingly demonstrated and remain active areas of research.

As a result of a greater understanding of the molecular pathogenesis of PV and MPNs, several novel agents are currently being tested in clinical trials for the treatment of PV. However, the search for new therapies that can halt the fibrotic or leukemic transformation of PV patients remains an urgent unmet need. In our opinion, the implementation of NGS techniques will continue to help clinicians in the accurate diagnosis of PV as well as to guide a more individualized clinical management in the future.

## Figures and Tables

**Table 1:** Summary of genetic differences between PV and ET that can help differentiate the two MPNs.

Molecular Marker	PV	ET
Homozygosity (or high allelic frequency) of *JAK2*V617F	More common	Less common
Mutations in *KMT2A* and/or *TP53*	More common	Less common
*NFE2* expression	Higher	Lower
HSP70 and calreticulin protein expression	Higher	Lower
miR-145 and/or miR-451 expression	Higher	Normal
miR-222 expression	Lower	Higher

**Table 2:** Stratification of risk of survival in polycythaemia vera according to the classical risk score.

Risk Factor	Score
Age ≥ 67 years	5
Age 57–66 years	2
Leukocytes ≥ 15 × 10^9^/L	1
Venous thrombosis	1

Low risk: score 0, intermediate risk: score 1 or 2, high risk: score ≥ 3 (adapted from [129]).

**Table 3:** Stratification of risk of survival in polycythaemia vera according to the Mutation-enhanced International Prognostic Scoring System (MIPSS-PV) (adapted from [130]).

Risk Factor	Score
Age ≥ 67 years	2
Leukocytes ≥ 15 × 10^9^/L	1
Thrombosis history	1
*SRSF2* mutation	3

**Table 4:** Stratification of risk of survival in post-PV MF according to the Myelofibrosis Secondary to PV and ET Prognostic Model (MYSEC-PM) (adapted from [181]). Patients are classified into low (score 0–10), intermediate 1 (score 11–13), intermediate 2 (score 14–15) or high (score > 16) risk groups. The MYSEC-PM is available online as a risk calculator (http://www.mysec-pm.eu/, accessed on 2 November 2020).

Risk Factor	Score
Haemoglobin < 11 g/dL	2
Circulating blasts ≥3%	2
*CALR*-unmutated ^1^	2
Platelets < 150 × 10^9^/L	1
Constitutional symptoms	1
Age	0.15 per year

^1^ For patients with post-ET MF.
